# *N*-(2,5-Dimeth­oxy­phen­yl)-6-nitro­quinazolin-4-amine

**DOI:** 10.1107/S1600536812048878

**Published:** 2012-12-05

**Authors:** Syed Muhammad Saad, Imran Khan, Shahnaz Perveen, Khalid M. Khan, Sammer Yousuf

**Affiliations:** a H.E.J. Research Institute of Chemistry, International Center for Chemical and Biological Sciences, University of Karachi, Karachi 75270, Pakistan; b PCSIR Labortories Complex, Karachi, Shahrah-e-Dr. Salmuzzaman Siddiqui, Karachi 75280, Pakistan

## Abstract

In the title mol­ecule, C_16_H_14_N_4_O_4_, the quinazoline ring is substanti­ally planar (r.m.s. deviation = 0.0129 Å) and forms a dihedral angle of 2.73 (8)° with the benzene ring. The conformation of the mol­ecule is stabilized by an intra­molecular C—H⋯N hydrogen bond. In the crystal, mol­ecules are linked into chains running parallel to the *b* axis by C—H⋯O hydrogen bonds. In addition, π–π stacking is observed between dimethoxy-substituted and nitro-substituted benzene rings, with centroid–centroid distances in the range 3.6438 (10)–3.7148 (10) Å.

## Related literature

For the biological activity of quinazoline derivatives, see: Arfan *et al.* (2008 ▶); Sheng-Li *et al.* (2005 ▶); Kung *et al.* (1999 ▶); Ram *et al.* (1990 ▶); Misra *et al.* (1981 ▶); Hess *et al.* (1968 ▶). For bond-length data, see: Allen *et al.* (1987 ▶).
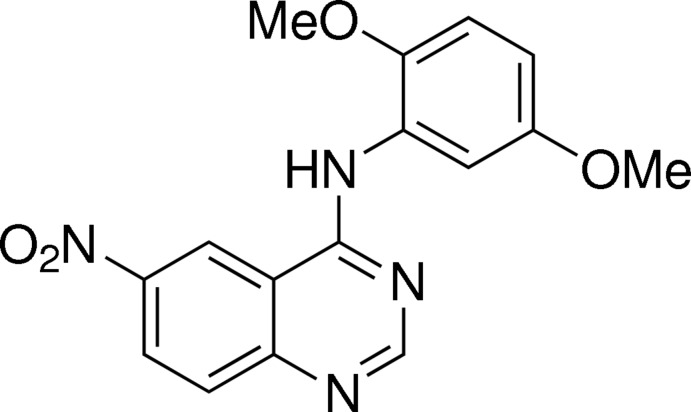


## Experimental

### 

#### Crystal data

C_16_H_14_N_4_O_4_
*M* *_r_* = 326.31
Triclinic, 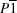

*a* = 7.2440 (7) Å
*b* = 10.2832 (10) Å
*c* = 11.1622 (11) Å
α = 72.475 (2)°
β = 83.663 (2)°
γ = 70.429 (2)°
*V* = 747.05 (13) Å^3^
*Z* = 2
Mo *K*α radiation
μ = 0.11 mm^−1^
*T* = 298 K
0.29 × 0.19 × 0.15 mm

#### Data collection

Bruker SMART APEX CCD area-detector diffractometer
Absorption correction: multi-scan (*SADABS*; Bruker, 2000 ▶) *T*_min_ = 0.970, *T*_max_ = 0.984
8510 measured reflections
2792 independent reflections
2249 reflections with *I* > 2σ(*I*)
*R*_int_ = 0.021

#### Refinement

*R*[*F* ^2^ > 2σ(*F* ^2^)] = 0.042
*wR*(*F* ^2^) = 0.120
*S* = 1.05
2792 reflections
224 parameters
H atoms treated by a mixture of independent and constrained refinement
Δρ_max_ = 0.18 e Å^−3^
Δρ_min_ = −0.20 e Å^−3^

### 

Data collection: *SMART* (Bruker, 2000 ▶); cell refinement: *SAINT* (Bruker, 2000 ▶); data reduction: *SAINT*; program(s) used to solve structure: *SHELXS97* (Sheldrick, 2008 ▶); program(s) used to refine structure: *SHELXL97* (Sheldrick, 2008 ▶); molecular graphics: *SHELXTL* (Sheldrick, 2008 ▶); software used to prepare material for publication: *SHELXTL*, *PARST* (Nardelli, 1995 ▶) and *PLATON* (Spek, 2009 ▶).

## Supplementary Material

Crystal structure: contains datablock(s) global, I. DOI: 10.1107/S1600536812048878/rz5029sup1.cif

Structure factors: contains datablock(s) I. DOI: 10.1107/S1600536812048878/rz5029Isup2.hkl

Supplementary material file. DOI: 10.1107/S1600536812048878/rz5029Isup3.cml

Additional supplementary materials:  crystallographic information; 3D view; checkCIF report

## Figures and Tables

**Table 1:** Hydrogen-bond geometry (Å, °)

*D*—H⋯*A*	*D*—H	H⋯*A*	*D*⋯*A*	*D*—H⋯*A*
C5—H5*A*⋯N2	0.93	2.22	2.833 (2)	123
C8—H8*A*⋯O3^i^	0.93	2.60	3.490 (2)	161

Symmetry code: (i) 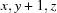.

**Fig. 1.**
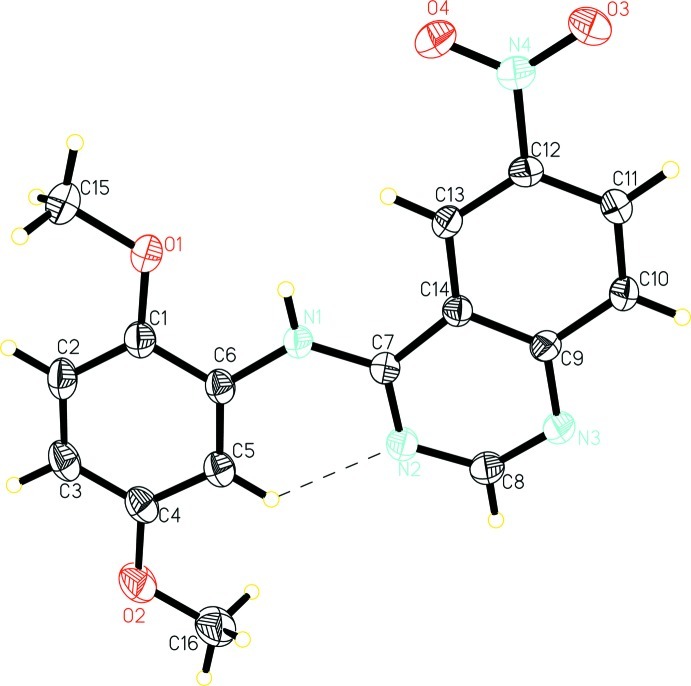
The molecular structure of the title compound with displacement ellipsoids drawn at the 30% probability level. An intramolecular hydrogen bond is shown as a dashed line.

**Fig. 2.**
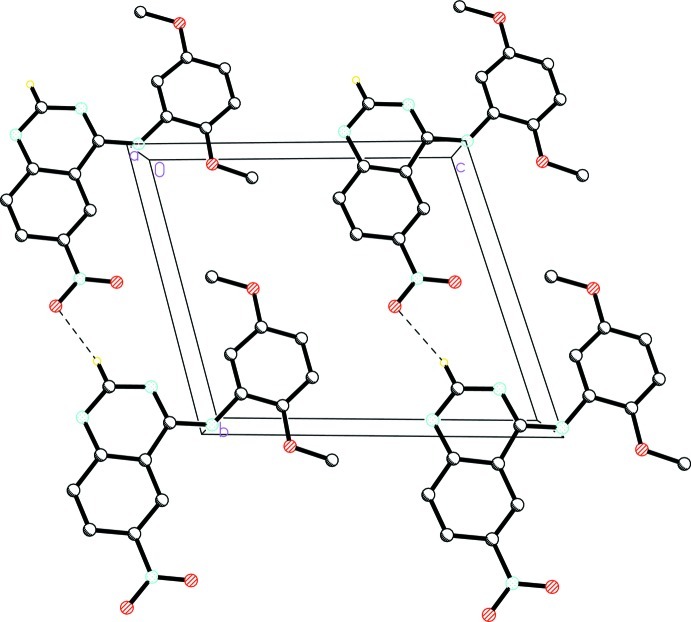
The crystal packing of the title compound. Only hydrogen atoms involved in hydrogen bonding (dashed lines) are shown.

## supplementary crystallographic information

### Comment

Quinazoline derivatives are the aromatic bicyclic compounds obtained upon the fusion of benzene and pyrimidine rings. Quinazoline analogs are found to be biologically active rendering a variety of therapeutic effects such as anti-inflammatory (Misra *et al.*, 1981), anticancer (Sheng-Li *et al.*, 2005), antihypertensive (Hess *et al.*, 1968), antibacterial (Kung *et al.*, 1999), leishmanicidal (Ram *et al.*, 1990) and antimicrobial (Arfan *et al.*, 2008) activities. The title compound is a quiazoline derivative synthesized as a part of our ongoing project in order to evaluate the biological potential of new derivatives of this important class of organic compounds.

The molecule of the title compound (Fig. 1) is composed of a phenyl (C1–C6) and a nearly planar quinazoline ring (N2–N3/C7–C14; r.m.s. deviation 0.0129 Å) linked through a C6—N1—C7 amino linkage. The bond lengths (Allen *et al.*, 1987) and angles were found to be in normal range. The molecular conformation is stabilized by an intramolecular C5—H5A···N2 hydrogen bond (Table 1). In the crystal, molecules form chains by intermolecular C8—H8A···O3 interactions (symmetry codes as in Table 1, Fig. 2) running parallel to the *b* axis. The crystal structure is further strengthened by significant π–π stackings: *Cg*(1)···*Cg*(2)^i^, 3.7148 (10) Å; *Cg*(1)···*Cg*(3)^i^, 3.7099 (10) Å; *Cg*(1)··· *Cg*(3)^ii^, 3.6438 (10) Å; *Cg*(1), *Cg*(2) and *Cg*(3) are the centroids of the C1–C6, N2/C7/C11/C9/N3/C8 and C9–C14 rings, respectively; symmetry codes: (i) 1-x, 2-y, -z; (ii) -x, 2-y, -z.

### Experimental

A mixture of (*E*)-*N*'-(2-cyano-4-nitrophenyl)-*N*,*N*-dimethylformimidamide (0.436 g, 2 mmol) and 2,5-dimethoxyaniline (0.35 g, 2 mmol) was refluxed in acetic acid (12 ml) at 75 °C for 2 h. Progress of the reaction was monitored by thin layer chromatography. After completion of the reaction, the mixture was cooled to room temperature and neutralized by adding a saturated aqueous solution of sodium bicarbonate until the evolution of CO_2_ gas ceased. The reaction mixture yielded brown crystals on standing at room temperature, which were filtered and washed with water. The crystals were re-grown using ethanol and collected in 50.2% yield (0.3273 g). All chemicals were purchased by sigma Aldrich Germany.

### Refinement

H atoms on aromatic rings and methyl carbons were positioned geomatrically with 0.93–0.96 Å and constrained to ride on their parent atoms with *U*_iso_(H) = 1.2 *U*_eq_(C) or 1.5 *U*_eq_(C) for methyl H atoms. The H atoms of the nitrogen atom (N–H = 0.868 (19) Å) was located in a difference Fourier map and refined isotropically. A rotating group model was applied to the methyl groups.

### Crystal data

**Table:**

C_16_H_14_N_4_O_4_	*Z* = 2
*M**_r_* = 326.31	*F*(000) = 340
Triclinic, *P*1	*D*_x_ = 1.451 Mg m^−^^3^
Hall symbol: -P 1	Mo *K*α radiation, λ = 0.71073 Å
*a* = 7.2440 (7) Å	Cell parameters from 2914 reflections
*b* = 10.2832 (10) Å	θ = 2.2–28.2°
*c* = 11.1622 (11) Å	µ = 0.11 mm^−^^1^
α = 72.475 (2)°	*T* = 298 K
β = 83.663 (2)°	Block, brown
γ = 70.429 (2)°	0.29 × 0.19 × 0.15 mm
*V* = 747.05 (13) Å^3^	

### Data collection

**Table:**

Bruker SMART APEX CCD area-detector diffractometer	2792 independent reflections
Radiation source: fine-focus sealed tube	2249 reflections with *I* > 2σ(*I*)
Graphite monochromator	*R*_int_ = 0.021
ω scan	θ_max_ = 25.5°, θ_min_ = 1.9°
Absorption correction: multi-scan (*SADABS*; Bruker, 2000)	*h* = −8→8
*T*_min_ = 0.970, *T*_max_ = 0.984	*k* = −12→12
8510 measured reflections	*l* = −13→13

### Refinement

**Table:**

Refinement on *F*^2^	Secondary atom site location: difference Fourier map
Least-squares matrix: full	Hydrogen site location: inferred from neighbouring sites
*R*[*F*^2^ > 2σ(*F*^2^)] = 0.042	H atoms treated by a mixture of independent and constrained refinement
*wR*(*F*^2^) = 0.120	*w* = 1/[σ^2^(*F*_o_^2^) + (0.0634*P*)^2^ + 0.1096*P*] where *P* = (*F*_o_^2^ + 2*F*_c_^2^)/3
*S* = 1.05	(Δ/σ)_max_ < 0.001
2792 reflections	Δρ_max_ = 0.18 e Å^−^^3^
224 parameters	Δρ_min_ = −0.20 e Å^−^^3^
0 restraints	Extinction correction: *SHELXL97* (Sheldrick, 2008), Fc^*^=kFc[1+0.001xFc^2^λ^3^/sin(2θ)]^-1/4^
Primary atom site location: structure-invariant direct methods	Extinction coefficient: 0.012 (3)

### Special details

**Table:**

Geometry. All e.s.d.'s (except the e.s.d. in the dihedral angle between two l.s. planes) are estimated using the full covariance matrix. The cell e.s.d.'s are taken into account individually in the estimation of e.s.d.'s in distances, angles and torsion angles; correlations between e.s.d.'s in cell parameters are only used when they are defined by crystal symmetry. An approximate (isotropic) treatment of cell e.s.d.'s is used for estimating e.s.d.'s involving l.s. planes.
Refinement. Refinement of *F*^2^ against ALL reflections. The weighted *R*-factor *wR* and goodness of fit *S* are based on *F*^2^, conventional *R*-factors *R* are based on *F*, with *F* set to zero for negative *F*^2^. The threshold expression of *F*^2^ > σ(*F*^2^) is used only for calculating *R*-factors(gt) *etc*. and is not relevant to the choice of reflections for refinement. *R*-factors based on *F*^2^ are statistically about twice as large as those based on *F*, and *R*- factors based on ALL data will be even larger.

### Fractional atomic coordinates and isotropic or equivalent isotropic displacement parameters (Å^2^)

**Table:**

	*x*	*y*	*z*	*U*_iso_*/*U*_eq_	
O1	0.32912 (17)	0.94268 (12)	−0.22927 (10)	0.0562 (3)	
O2	0.3236 (2)	1.48679 (13)	−0.24856 (12)	0.0691 (4)	
O3	0.1791 (2)	0.42539 (13)	0.34841 (13)	0.0830 (5)	
O4	0.2239 (2)	0.50964 (14)	0.15126 (12)	0.0760 (4)	
N1	0.25382 (19)	1.01670 (14)	−0.02167 (11)	0.0433 (3)	
H1A	0.271 (2)	0.936 (2)	−0.0378 (16)	0.057 (5)*	
N2	0.1798 (2)	1.14710 (13)	0.12319 (12)	0.0509 (4)	
N3	0.1201 (2)	1.04595 (13)	0.34070 (12)	0.0490 (3)	
N4	0.1950 (2)	0.52243 (14)	0.25729 (13)	0.0527 (4)	
C1	0.3302 (2)	1.07924 (17)	−0.23984 (14)	0.0458 (4)	
C2	0.3659 (2)	1.1756 (2)	−0.34973 (15)	0.0538 (4)	
H2B	0.3923	1.1489	−0.4239	0.065*	
C3	0.3625 (2)	1.31047 (19)	−0.35023 (15)	0.0553 (4)	
H3B	0.3870	1.3742	−0.4244	0.066*	
C4	0.3231 (2)	1.35085 (17)	−0.24107 (15)	0.0498 (4)	
C5	0.2860 (2)	1.25646 (16)	−0.12990 (15)	0.0470 (4)	
H5A	0.2590	1.2843	−0.0563	0.056*	
C6	0.2894 (2)	1.12074 (16)	−0.12903 (13)	0.0413 (3)	
C7	0.2062 (2)	1.02516 (15)	0.09734 (13)	0.0383 (3)	
C8	0.1392 (3)	1.14894 (17)	0.24328 (15)	0.0543 (4)	
H8A	0.1223	1.2361	0.2589	0.065*	
C9	0.1398 (2)	0.91960 (15)	0.31559 (13)	0.0388 (3)	
C10	0.1148 (2)	0.80460 (16)	0.41591 (14)	0.0444 (4)	
H10A	0.0859	0.8167	0.4959	0.053*	
C11	0.1322 (2)	0.67639 (16)	0.39744 (14)	0.0440 (4)	
H11A	0.1156	0.6007	0.4638	0.053*	
C12	0.1754 (2)	0.66082 (14)	0.27654 (14)	0.0399 (3)	
C13	0.2004 (2)	0.76883 (15)	0.17581 (13)	0.0388 (3)	
H13A	0.2281	0.7546	0.0965	0.047*	
C14	0.18346 (19)	0.90156 (14)	0.19406 (13)	0.0358 (3)	
C15	0.3816 (3)	0.8934 (2)	−0.33839 (17)	0.0663 (5)	
H15A	0.3811	0.7959	−0.3187	0.099*	
H15B	0.5102	0.8974	−0.3664	0.099*	
H15C	0.2889	0.9534	−0.4036	0.099*	
C16	0.2820 (3)	1.5289 (2)	−0.1356 (2)	0.0781 (6)	
H16A	0.2891	1.6242	−0.1515	0.117*	
H16B	0.3760	1.4631	−0.0730	0.117*	
H16C	0.1528	1.5278	−0.1061	0.117*	

### Atomic displacement parameters (Å^2^)

**Table:**

	*U*^11^	*U*^22^	*U*^33^	*U*^12^	*U*^13^	*U*^23^
O1	0.0745 (8)	0.0609 (7)	0.0399 (6)	−0.0283 (6)	0.0097 (5)	−0.0198 (5)
O2	0.0911 (9)	0.0502 (7)	0.0614 (8)	−0.0316 (7)	0.0063 (7)	−0.0012 (6)
O3	0.1479 (14)	0.0451 (7)	0.0626 (8)	−0.0462 (8)	0.0084 (8)	−0.0107 (6)
O4	0.1248 (12)	0.0602 (8)	0.0589 (8)	−0.0407 (8)	0.0150 (8)	−0.0326 (6)
N1	0.0579 (8)	0.0396 (7)	0.0338 (7)	−0.0191 (6)	0.0037 (5)	−0.0098 (5)
N2	0.0736 (9)	0.0398 (7)	0.0417 (7)	−0.0227 (6)	0.0058 (6)	−0.0123 (6)
N3	0.0688 (9)	0.0436 (7)	0.0419 (7)	−0.0235 (6)	0.0078 (6)	−0.0189 (6)
N4	0.0701 (9)	0.0434 (7)	0.0505 (8)	−0.0224 (7)	0.0030 (7)	−0.0181 (6)
C1	0.0430 (8)	0.0549 (9)	0.0386 (8)	−0.0169 (7)	0.0003 (6)	−0.0105 (7)
C2	0.0529 (9)	0.0717 (11)	0.0335 (8)	−0.0209 (8)	0.0025 (7)	−0.0098 (7)
C3	0.0542 (10)	0.0614 (10)	0.0399 (9)	−0.0218 (8)	−0.0014 (7)	0.0048 (7)
C4	0.0484 (9)	0.0474 (9)	0.0461 (9)	−0.0165 (7)	−0.0019 (7)	−0.0003 (7)
C5	0.0495 (9)	0.0470 (8)	0.0405 (8)	−0.0164 (7)	0.0007 (7)	−0.0060 (7)
C6	0.0394 (8)	0.0469 (8)	0.0336 (8)	−0.0143 (6)	−0.0016 (6)	−0.0048 (6)
C7	0.0402 (8)	0.0403 (8)	0.0349 (7)	−0.0138 (6)	0.0008 (6)	−0.0109 (6)
C8	0.0794 (12)	0.0412 (8)	0.0498 (9)	−0.0251 (8)	0.0078 (8)	−0.0199 (7)
C9	0.0414 (8)	0.0414 (8)	0.0374 (8)	−0.0154 (6)	0.0023 (6)	−0.0152 (6)
C10	0.0564 (9)	0.0474 (8)	0.0331 (7)	−0.0210 (7)	0.0079 (6)	−0.0146 (6)
C11	0.0518 (9)	0.0432 (8)	0.0375 (8)	−0.0204 (7)	0.0040 (6)	−0.0078 (6)
C12	0.0441 (8)	0.0357 (7)	0.0430 (8)	−0.0140 (6)	0.0001 (6)	−0.0140 (6)
C13	0.0444 (8)	0.0410 (8)	0.0334 (7)	−0.0137 (6)	0.0012 (6)	−0.0142 (6)
C14	0.0359 (7)	0.0377 (7)	0.0341 (7)	−0.0119 (6)	0.0007 (6)	−0.0105 (6)
C15	0.0800 (13)	0.0786 (12)	0.0517 (10)	−0.0321 (10)	0.0150 (9)	−0.0323 (9)
C16	0.1048 (16)	0.0540 (11)	0.0800 (14)	−0.0356 (11)	0.0166 (12)	−0.0196 (10)

### Geometric parameters (Å, º)

**Table:**

O1—C1	1.3761 (19)	C4—C5	1.389 (2)
O1—C15	1.4234 (19)	C5—C6	1.385 (2)
O2—C4	1.376 (2)	C5—H5A	0.9300
O2—C16	1.423 (2)	C7—C14	1.4430 (19)
O3—N4	1.2179 (17)	C8—H8A	0.9300
O4—N4	1.2176 (17)	C9—C10	1.409 (2)
N1—C7	1.3564 (18)	C9—C14	1.4115 (19)
N1—C6	1.4112 (18)	C10—C11	1.358 (2)
N1—H1A	0.868 (19)	C10—H10A	0.9300
N2—C7	1.3182 (19)	C11—C12	1.396 (2)
N2—C8	1.345 (2)	C11—H11A	0.9300
N3—C8	1.302 (2)	C12—C13	1.367 (2)
N3—C9	1.3687 (18)	C13—C14	1.4033 (19)
N4—C12	1.4608 (18)	C13—H13A	0.9300
C1—C2	1.387 (2)	C15—H15A	0.9600
C1—C6	1.397 (2)	C15—H15B	0.9600
C2—C3	1.377 (2)	C15—H15C	0.9600
C2—H2B	0.9300	C16—H16A	0.9600
C3—C4	1.374 (2)	C16—H16B	0.9600
C3—H3B	0.9300	C16—H16C	0.9600
			
C1—O1—C15	117.06 (13)	N3—C8—H8A	115.4
C4—O2—C16	116.73 (13)	N2—C8—H8A	115.4
C7—N1—C6	129.76 (13)	N3—C9—C10	117.95 (13)
C7—N1—H1A	118.8 (12)	N3—C9—C14	122.40 (13)
C6—N1—H1A	111.4 (12)	C10—C9—C14	119.65 (12)
C7—N2—C8	117.26 (13)	C11—C10—C9	120.95 (13)
C8—N3—C9	114.77 (13)	C11—C10—H10A	119.5
O4—N4—O3	123.00 (13)	C9—C10—H10A	119.5
O4—N4—C12	118.81 (13)	C10—C11—C12	118.47 (13)
O3—N4—C12	118.18 (13)	C10—C11—H11A	120.8
O1—C1—C2	125.36 (14)	C12—C11—H11A	120.8
O1—C1—C6	115.44 (13)	C13—C12—C11	123.04 (13)
C2—C1—C6	119.20 (15)	C13—C12—N4	118.71 (13)
C3—C2—C1	120.74 (15)	C11—C12—N4	118.25 (13)
C3—C2—H2B	119.6	C12—C13—C14	118.91 (13)
C1—C2—H2B	119.6	C12—C13—H13A	120.5
C4—C3—C2	119.96 (14)	C14—C13—H13A	120.5
C4—C3—H3B	120.0	C13—C14—C9	118.98 (12)
C2—C3—H3B	120.0	C13—C14—C7	125.30 (12)
C3—C4—O2	116.71 (14)	C9—C14—C7	115.72 (12)
C3—C4—C5	120.38 (15)	O1—C15—H15A	109.5
O2—C4—C5	122.91 (15)	O1—C15—H15B	109.5
C6—C5—C4	119.81 (15)	H15A—C15—H15B	109.5
C6—C5—H5A	120.1	O1—C15—H15C	109.5
C4—C5—H5A	120.1	H15A—C15—H15C	109.5
C5—C6—C1	119.91 (13)	H15B—C15—H15C	109.5
C5—C6—N1	124.48 (13)	O2—C16—H16A	109.5
C1—C6—N1	115.61 (13)	O2—C16—H16B	109.5
N2—C7—N1	119.40 (13)	H16A—C16—H16B	109.5
N2—C7—C14	120.64 (13)	O2—C16—H16C	109.5
N1—C7—C14	119.95 (12)	H16A—C16—H16C	109.5
N3—C8—N2	129.14 (14)	H16B—C16—H16C	109.5
			
C15—O1—C1—C2	4.4 (2)	C7—N2—C8—N3	−0.5 (3)
C15—O1—C1—C6	−176.33 (14)	C8—N3—C9—C10	−178.01 (14)
O1—C1—C2—C3	179.65 (14)	C8—N3—C9—C14	1.9 (2)
C6—C1—C2—C3	0.4 (2)	N3—C9—C10—C11	179.96 (14)
C1—C2—C3—C4	−0.2 (2)	C14—C9—C10—C11	0.0 (2)
C2—C3—C4—O2	179.56 (14)	C9—C10—C11—C12	0.0 (2)
C2—C3—C4—C5	−0.1 (2)	C10—C11—C12—C13	−0.2 (2)
C16—O2—C4—C3	179.79 (16)	C10—C11—C12—N4	179.70 (13)
C16—O2—C4—C5	−0.6 (2)	O4—N4—C12—C13	−3.3 (2)
C3—C4—C5—C6	0.2 (2)	O3—N4—C12—C13	177.54 (14)
O2—C4—C5—C6	−179.49 (14)	O4—N4—C12—C11	176.73 (14)
C4—C5—C6—C1	0.1 (2)	O3—N4—C12—C11	−2.4 (2)
C4—C5—C6—N1	−179.94 (13)	C11—C12—C13—C14	0.5 (2)
O1—C1—C6—C5	−179.67 (13)	N4—C12—C13—C14	−179.45 (12)
C2—C1—C6—C5	−0.4 (2)	C12—C13—C14—C9	−0.5 (2)
O1—C1—C6—N1	0.34 (19)	C12—C13—C14—C7	179.88 (13)
C2—C1—C6—N1	179.66 (13)	N3—C9—C14—C13	−179.70 (13)
C7—N1—C6—C5	1.8 (2)	C10—C9—C14—C13	0.2 (2)
C7—N1—C6—C1	−178.23 (14)	N3—C9—C14—C7	0.0 (2)
C8—N2—C7—N1	−177.84 (14)	C10—C9—C14—C7	179.89 (12)
C8—N2—C7—C14	2.6 (2)	N2—C7—C14—C13	177.34 (13)
C6—N1—C7—N2	2.7 (2)	N1—C7—C14—C13	−2.2 (2)
C6—N1—C7—C14	−177.71 (13)	N2—C7—C14—C9	−2.3 (2)
C9—N3—C8—N2	−1.8 (3)	N1—C7—C14—C9	178.10 (12)

### Hydrogen-bond geometry (Å, º)

**Table:**

*D*—H···*A*	*D*—H	H···*A*	*D*···*A*	*D*—H···*A*
C5—H5*A*···N2	0.93	2.22	2.833 (2)	123
C8—H8*A*···O3^i^	0.93	2.60	3.490 (2)	161

Symmetry code: (i) *x*, *y*+1, *z*.
